# Engagement by Design: Belongingness, Cultural Value Orientations, and Pathways into Emerging Technologies

**DOI:** 10.3390/bs15101358

**Published:** 2025-10-05

**Authors:** Daisuke Akiba, Michael Perrone, Caterina Almendral, Rebecca Garte

**Affiliations:** 1 Graduate Center, The City University of New York, 365 Fifth Avenue, New York, NY 10016, USA; 2 Queens College, The City University of New York, Queens, NY 11367, USA; michael.perrone@qc.cuny.edu; 3 LaGuardia Community College, The City University of New York, Long Island City, NY 11101, USA; calmendral@lagcc.cuny.edu; 4 Borough of Manhattan Community College, The City University of New York, New York, NY 10007, USA; rgarte@bmcc.cuny.edu

**Keywords:** belonging, belongingness, collectivism, cultural value orientations, culturally responsive computing, digital inclusion, educational equity, horizontal collectivism, STEM equity, technology achievement gaps, technology engagement, vertical collectivism

## Abstract

This theoretical article examines how belongingness, defined as the sense that one’s participation is legitimate and valued, interacts with cultural value orientations to help explain persistent disparities in U.S. technology engagement, including emerging technologies, across racial and ethnic groups. While structural barriers (e.g., racism, poverty, linguistic bias, etc.) remain essential to understanding such inequity, we argue that engagement patterns in technology also reflect how different cultural communities may define and experience belongingness in relation to digital domains. Drawing on Triandis and Gelfand’s framework, and focusing specifically on educational contexts, we propose the Belongingness through Cultural Value Alignment (BCVA) model, whereby belongingness serves as a catalyst between cultural value orientations and technology engagement, with vertical collectivism deriving belongingness primarily through structured skill development and validation while horizontal collectivism focusing instead on belonging based on community integration. When technological environments value practices that are consistent with vertical collectivist norms, individuals from horizontal collectivist cultures may experience cultural misalignment not from disinterest in technology or exclusionary efforts but, instead, because dominant engagement modes conflict with their familiar frameworks for fostering a sense of belonging. By examining how cultural value orientations mediate the sense of belonging in contexts involving modern technologies, the proposed perspective offers a novel framework for understanding why access alone may have proven insufficient to address technological participation gaps, and suggests directions for creating technology spaces where individuals from a wider range of communities can experience the authentic sense of belonging.

## 1. Introduction

Persistent disparities in technology achievement across racial and ethnic groups in the United States remain a pressing challenge, evident in completion rates in computer science and other STEM programs, representation in careers in emerging technologies, and advancement to technology leadership (Whitacre et al., 2023b). Structural barriers (e.g., racism, economic inequality, and linguistic mismatch) have widely been cited as the primary cause underlying these disparities, but they do not fully account for differential patterns of technology engagement. For example, the high levels of participation and achievement among many Asian American communities in the technology spaces, despite histories of racism, economic constraints, and language barriers, point to limits of purely structural accounts (Hsin & Xie, 2014; Varma, 2023). To address these complexities, this theoretical article is grounded in Triandis and Gelfand’s (1998) framework and proposes the Belongingness through Cultural Value Alignment (BCVA) model, which holds that context-relevant cultural value orientations shape how belongingness is fostered in technology spaces, thereby influencing participation and achievement. We examine disparities through the lens of educational and training experiences, considering how common pedagogies, assignment structures, and evaluation systems may differentially cue validation and belongingness across cultural orientations. As a theory paper, we analyze no new data; instead, our goal is to articulate a testable model and derive design-relevant guidance.

In addition, for clarity, this analysis defines “technology engagement” and its derivatives as encompassing two interconnected dimensions: (1) sustained and successful participation in formal technology education and training, including computing-related classes, computing and digital literacies education and professional development, to advanced degrees, and (2) pursuit of skills and careers in emerging technologies that emphasize design, innovation, and solution-building rather than consumption or marketing. This definition deliberately focuses on pathways toward active technology leadership and innovation, recognizing that, while many communities demonstrate enthusiasm for technology use, persistent gaps remain in who creates the technologies that shape our digital future. We contend that, in addition to the traditional explanations introduced below, these dimensions of technology engagement intersect with how members of the communities cultivate and apply computing and digital literacies, which are increasingly critical for equitable participation in emerging technology spaces and careers. We therefore frame belongingness as the cultural bridge linking value orientations to engagement, underscoring the need for culturally aligned CDL education and training to support learners from a wide range of cultural backgrounds.

Despite increased investments in access and infrastructure, Black and Latino learners and professionals in the U.S. remain significantly underrepresented in technology-related domains (e.g., software engineering, hardware development, data science, cybersecurity, and systems architecture) compared to their White and Asian/Asian American counterparts (Darling-Hammond, 2010; Fry et al., 2021). Traditional accounts of these disparities have primarily emphasized systemic inequities, such as White supremacy, racism, and economic deprivation (e.g., digital divide) between those *with* and *without* adequate technology access (Calaza et al., 2021; Whitacre et al., 2023a). Critical Race Theory (CRT), in particular, has illuminated how entrenched power structures and racial hierarchies shape educational and technological interest and opportunities (Ladson-Billings & TateIv, 1995; Dixson & Rousseau Anderson, 2018). While these structural factors are undoubtedly indispensable for understanding broader inequities, they may not fully explain variation in achievement patterns among communities with experiences of marginalization. Such barriers are not only material (i.e., access to goods and services) but also psychological, influencing individuals’ perceptions of legitimacy, fit, and belonging in educational and technological environments (Boyd et al., 2024; Godwin et al., 2024).

While these socioeconomic and other systemic factors may certainly contribute to inequitable educational and professional outcomes in the technological domains across racial/ethnic groups, they have also faced an explanatory challenge: they cannot convincingly account for the relative success of some groups, such as Asian Americans, in technology fields, despite the lengthy history of systemic discrimination in the U.S., the collective experience of poverty, and linguistic challenges. In addition to the routine experiences of anti-Asian racism in the U.S. (Yu et al., 2024), Asian Americans have faced systemic, government-led barriers since the early history of the United States, including school segregation, various iterations of the Chinese Exclusion Act of 1882 and similar immigration restrictions from Asian nations to the U.S. that persisted through much of the 1960s (Hsu, 2015), as well as the mass incarceration of Japanese Americans (Ogawa, 2004). Perhaps more counterintuitively, publicly available data indicate that Asian Americans, on average, experience poverty rates comparable to and, in several major metropolitan areas like New York and Los Angeles, higher than, Black or Latino communities (Tran, 2018; Hill, 2023; Boyd et al., 2024). These facts diametrically contradict the widespread misconception that Asian Americans are: (a) newcomers who have never faced systemic racism in the U.S., and (b) generally affluent because immigration from Asian nations is primarily limited to those of higher socioeconomic status.

Similarly, linguistic mismatch (i.e., standard American English vs. Black American English or Spanish and its derivatives, such as *Spanglish*—a seamless mix of Spanish and English) has frequently been cited as one of the primary barriers to Black and Latino students’ academic performance in the U.S. (Smith et al., 2019; Mallinson, 2024). Yet Asian American students often achieve strong academic outcomes despite facing significant English language barriers (G. Li et al., 2023). Furthermore, even when individuals have equal access to technology, engagement outcomes often remain unequal across racial/ethnic groups, suggesting that access alone is not enough to ensure a sense of belonging, let alone authentic and sustainable participation (Scott et al., 2013). While we acknowledge that structural factors remain vital for understanding technology disparities, this article proposes a complementary framework examining how cultural value orientations shape the experience of belongingness in technology spaces, which in turn catalyzes differential patterns of engagement and achievement. Specifically, we explore how vertical and horizontal collectivist orientations create distinct pathways to belonging that may explain why some communities thrive in current technology education structures while others, despite equal interest and capability, remain underrepresented in technology production roles. This analysis moves beyond access to examine the psychological mechanisms linking cultural frameworks to technology participation.

Emerging research, incidentally, suggests that access alone does not guarantee meaningful engagement in technology fields. Instead, students’ “sense of belonging” or “belongingness,” both defined as the subjective feeling of fit and acceptance, may serve as a critical psychological factor linking structural opportunity and actual engagement (Yi et al., 2024). This insight tackles a key limitation in conventional structure-focused frameworks, which have long emphasized systemic oppression (including Eurocentric curricula), economic hardship, and linguistic mismatch as the primary drivers of academic disparities (Dixson & Rousseau Anderson, 2018). While these structural factors remain vital, decades of well-intentioned intervention efforts targeting them have yielded uneven and modest improvements, if at all, in achievement outcomes (Gouvea, 2021). These inconsistencies suggest the need to reconsider the assumption that Asian Americans have uniquely bypassed structural barriers, allowing them to thrive in technological spaces where, according to structure-focused perspectives, they are theoretically excluded (Zou & Cheryan, 2017). Instead, we contend that we could potentially utilize a more nuanced framework, which would recognize how belongingness itself may be differentially shaped by cultural expectations and orientations, even when faced with substantively similar sets of challenges. Though belonging may be a universal human need (J. Li et al., 2025), the way it is experienced, fostered, interpreted, and expressed may vary significantly across cultural communities, particularly when those communities’ value orientations are misaligned with dominant institutional norms. By understanding how these orientations influence the sense of belonging, therefore, we may be better positioned to design educational and technological spaces that foster authentic engagement across diverse populations. Now, how is the sense of belonging fostered and explained in the technological spaces?

We propose that cultural value orientations (Hofstede, 1980) offer a compelling lens for interpreting how diverse communities experience belonging in technology spaces. The ostensible “Asian American exception,” for example, complicates explanations that rely solely on structural barriers such as White supremacy, poverty, or linguistic mismatch. Rather than discounting these forces, this pattern points to the need for frameworks that can illuminate how different cultural groups perceive, negotiate, and sustain belonging in contexts structured by the dominant cultural norms and expectations. Although one prior study attempted to examine cultural value orientations in technology-mediated learning contexts (Hornik & Tupchiy, 2006), this work suffered from significant conceptual and methodological limitations, including a reductive treatment of culture as fixed individual traits and reliance on survey-based cultural categorization that failed to capture the dynamic, contextual nature of technology use. Therefore, rather than building upon this foundation, the current framework takes a fundamentally different approach by examining how cultural orientations shape the experience of belongingness itself within technology spaces.

While structural barriers undoubtedly constrain opportunity, psychological factors—particularly the sense of belonging—play a crucial role in translating access into sustained engagement. Baumeister and Leary’s (1995) foundational work establishes belonging as a fundamental human motivation that drives behavior across contexts. In educational settings, Gopalan and Brady (2020) revealed that students’ sense of belonging correlates more strongly with academic persistence than socioeconomic factors alone. Similarly, within technology domains, Good et al. (2012) posited that belonging interventions increased women’s motivation to pursue computer science careers, while Walton and Cohen’s (2007) studies showed that addressing belonging uncertainty improved STEM performance among underrepresented minorities. These findings suggest that belongingness serves not merely as a pleasant addition to learning environments but as a critical psychological catalyst that transforms cultural orientations into active engagement patterns, making it essential to understand how different communities construct and experience belonging in technology spaces.

To that end, this analysis draws on Triandis and Gelfand’s (1998) framework of cultural value orientations to examine how people from collectivist backgrounds may pursue belonging in ways shaped by community norms. Specifically, we ask: How might different forms of collectivism, specifically vertical versus horizontal, impact pathways to belonging, and how might this lens complement structural analyses of participation gaps among minoritized U.S. communities in the technological domains?

Some disclaimers are warranted before proceeding. First, it needs to be emphasized early on that cultural orientations vary widely within communities, and the framework offered here is intended to highlight the general, overall tendencies across groups, rather than narrowly defined definitive traits each group carries. Needless to say, therefore, cultural value orientations are context-linked tendencies, not group essences (Hussain et al., 2021), as explained in further detail in later sections. Similarly, while factors such as gender, religion, or family structure intersect with cultural value orientations, our current analysis places its focus on collectivist orientations as they have been established in existing literature (Barton & Tan, 2010). The article centers Black, Latino, and Asian/Asian American communities, each of which exhibits collectivist *tendencies*, perhaps in different ways. White Americans and individualist frameworks, thus, are intentionally omitted here as a strategic delimitation to sharpen the current exploratory inquiry into the ways through which the sense of belonging might be fostered and experienced by the members of America’s major minoritized racial/ethnic groups. We acknowledge significant heterogeneity within these broad categories (e.g., East Asian experiences differ markedly from South Asian ones, Caribbean Black communities bring different cultural frameworks than African American ones, and Mexican American patterns may diverge from those of Puerto Rican or Central American communities). This initial theoretical scaffolding necessarily simplifies complex realities to establish a foundational framework; future research should examine how these within-group variations intersect with the broad patterns we identify here.

Additionally, throughout this article, the terms “Asian” or “Asian American” follow conventional U.S. usage, encompassing individuals of East, South, and Southeast Asian descent (e.g., Chinese, Indian, Japanese, Korean, etc.). This differs from the common British English usage, where “Asian” often refers primarily to individuals of South Asian heritage. While this article focuses on U.S.-based patterns, the conceptual framework we develop offers broader insight into how cultural orientations shape educational and technological engagement worldwide. We invite future research to explore these dynamics across global contexts, as discussed in more detail toward the end of this manuscript.

In short, this article emphasizes cultural value orientations to shed light on underexplored cultural community-level patterns that may exert influence on engagement with technology spaces (e.g., sustained participation in coding bootcamps, completion of technology degrees, pursuit of tech internships, and transition from technology consumption to creation), and the intent here is not, in any way, to essentialize cultural groups or to downplay the impact of structural biases and challenges. By emphasizing cultural strengths of all groups, particularly those that diverge greatly from the dominant norms, this framework avoids deficit narratives pervasive in discussions of minoritized populations (as pointed out by Gouvea, 2021 and Ling et al., 2025). Ultimately, we aim to supplement structural critiques with a theoretically grounded account of how cultural value orientations may contribute to the experience of belonging, and thereby engagement, achievement, and inclusion in technological domains (Godwin et al., 2024). The remainder of this article demonstrates how this *belongingness-as-a-catalyst framework* explains technology engagement patterns and suggests concrete pathways for educational intervention.

### Cultural Value Orientations: Beyond the Individualism–Collectivism Dichotomy

Research has traditionally positioned collectivism and individualism as opposites, associating Western societies with individualism and non-Western societies with collectivism (Hofstede, 1980). Yet this binary proved too simplistic to capture the nuanced ways people relate to groups and authority across cultures. In response, Triandis and Gelfand (1998) refined the framework by introducing vertical and horizontal dimensions to both collectivism and individualism. In the context of this paper, the collectivist variants (i.e., vertical collectivism [VC] and horizontal collectivism [HC]) are particularly relevant for understanding cultural belongingness among minoritized U.S. groups. These orientations entail fundamentally different expectations and emphases of group dynamics: VC emphasizes hierarchy, duty, and respect for expert figures, while HC stresses egalitarianism, interdependence, and communal solidarity (Moon et al., 2018). Previous research has consistently illustrated that these two forms of collectivism are not randomly distributed but, instead, tend to cluster along cultural lines. VC is especially prevalent in many Asian societies and among Asian Americans with roots in these societies, where group belonging typically entails respect for elders, authority figures, institutional hierarchies, and achievement-oriented norms (R. E. Nisbett et al., 2001; R. Nisbett, 2004; Moon et al., 2018). In contrast, HC has been associated more frequently with Black (Komarraju & Cokley, 2008) and Latino (Hussain et al., 2021) communities in the United States, where group belongingness is likely built and fostered through peer solidarity, egalitarianism, mutual camaradery, and community-based equality (Vélez-Ibáñez & Greenberg, 1992).

It would be remiss not to mention here that, despite these profoundly influential studies, analysis by Vargas and Kemmelmeier (2013) revealed no clear and consistent *quantitative* differences in collectivism levels (i.e., the magnitude to which individuals subscribe to ideas pertaining to collectivism) among ethnic groups in the United States, which may raise important questions about frameworks based on cultural value orientations. However, it should be emphasized that the current analysis focuses not on absolute levels of collectivism but on how these qualitatively divergent orientations may manifest within technology spaces in relative terms across groups. We therefore examine tendencies in how communities engage with technological domains rather than claiming essential cultural differences, recognizing that technology spaces themselves are culturally constructed environments that may resonate differently with varying value emphases.

While broad generalizations always risk oversimplification, the framework outlined here aims to capture common collectivist tendencies often found across Black, Latino, and Asian/Asian American communities. It is not intended as a universal model, nor does it apply to groups primarily shaped by individualist cultural orientations. Rather, it offers a culturally grounded lens to understand how different communities cultivate belonging through distinct modes of socialization, group structure, interaction, and knowledge-sharing, each of which influences how individuals engage with educational systems and technology domains. Table 1 presents a comparative framework highlighting how belongingness may be differently conceptualized and experienced within VC and HC contexts. Derived from the works by Triandis and Gelfand (1998), R. E. Nisbett et al. (2001), and R. Nisbett (2004), this illustrates and contrasts these orientations across key structural, relational, and process-based dimensions relevant to technology engagement.

Building on Table 1, we can focus on how these relative contrasts operate through belongingness. In technology-intensive settings, these orientations may shape participation, legitimacy, and recognition. We therefore treat them as context-activated tendencies and dynamics, rather than rigid categories. This framing aims to help explain differential engagement in technology domains.

Belongingness as a key element. While cultural value orientations shape the general tendencies of engagement, belongingness—or a sense of belonging—plays a critical role in determining how individuals respond to specific environments. Belongingness acts as a psychological lens through which individuals interpret the relevance, legitimacy, and accessibility of participation (Boyd et al., 2024). In both VC and HC contexts, the *desire to belong* should motivate technology engagement; however, the pathways may differ. For VC learners, as noted above, belonging is often affirmed through performance within structured hierarchies: formal recognition, academic and professional achievement, or expert validation. By contrast, HC learners often experience belonging through peer recognition, community affirmation, and contributions to collective well-being of the community members. In that sense, focusing on the sense of belonging helps “translate” broader emphases in cultural value orientations into personally meaningful engagement. The proposed model, therefore, considers belongingness as a process rather than an outcome leading to technological engagement. It should be noted here that belongingness is not norm-neutral; rather, what constitutes “legitimate participation” is contextually produced through such means as classroom practices and policies (Barton & Tan, 2010; Nasir & Hand, 2008).

When the demands and expectations of the technology spaces align with these cultural expectations, whether by emphasizing collaborative contribution or rewarding hierarchical achievement, we argue that they can foster a sense of fit, authenticity, and sustained participation for individuals with varying cultural value orientations. However, when dominant norms in tech spaces conflict with these cultural priorities, individuals may theoretically feel out of place or disenfranchised, even when they have equal access to support and resources (Godwin et al., 2024). Thus, belongingness offers a vital interpretive framework for understanding how cultural orientations become lived patterns of engagement, persistence, or withdrawal in technology contexts. This understanding opens the door to a more intentional and equity-driven approach.

Taken together, these insights point to a critical opportunity, as follows: by centering the sense of belonging, not as an incidental outcome but as a foundational motivator for technology engagement, we can more effectively support Black and brown learners and professionals. Our proposal, therefore, is to treat belonging as a *catalyst*, one that ignites and sustains engagement when learning environments resonate with the cultural value orientations of the communities we aim to serve. In practice, this means designing technology learning spaces that affirm peer-based solidarity and egalitarian contribution for HC learners, while also supporting structure, mentorship, and expert recognition for those from VC backgrounds. Rather than assuming a one-size-fits-all model of belonging and engagement, therefore, we argue for a responsive, culturally grounded approach where belonging is not simply one of the “nice-to-have” elements but, instead, essential to educational equity in the digital age.

## 2. Technology Engagement Across Cultural Value Orientations

Having established belongingness as a key catalyst between cultural orientations and technology engagement, we now examine how VC and HC frameworks create distinct belongingness experiences that drive different patterns of technology participation. The following analysis demonstrates how belongingness, not just cultural preference, connects cultural orientation and active technology engagement. The following sections explore the specific patterns of technology integration that emerge within these different cultural frameworks, highlighting how they influence academic and professional pathways in technology fields.

Vertical Collectivist (VC) Technology Integration. VC creates distinctive patterns in technology integration that align with academic achievement in technology-related domains. These include:

1. Expert-sanctioned learning and hierarchical competence development: VC cultures across Asia and Asian diaspora demonstrate learning traditions characterized by what J. Li (2012) calls a “virtue orientation” towards knowledge acquisition. This approach emphasizes methodical and persistent knowledge and skill development through disciplined pathways, respect for established expertise hierarchies, and structured progression. It would be feasible to argue that VC shapes technology integration by positioning technologies within hierarchical frameworks, where both technologies and learners occupy defined roles. The emphasis on comprehensive mastery and progression creates a natural alignment with design and algorithms in technological landscape, which similarly requires hierarchical knowledge structures. Individuals from these contexts develop cognitive frameworks emphasizing structured and integrated problem-solving (R. E. Nisbett et al., 2001), facilitating the precise rule application central to technology domains like programming, algorithms, and systems design. This alignment helps explain the *strong* representation of individuals from VC cultures in technology fields.
2. Role-based integration: As noted above, technologies are positioned within existing hierarchical role structures, with clear expectations about their functions and limitations. This also creates cognitive comfort with technologies as specialized contributors within existing social structures (Kim & Sherman, 2007). This orderly approach to defining relationships between components, thus, directly parallels programming paradigms, database design, and systems architecture, all of which depend on precisely organized hierarchical structures and clear role definitions.
3. Convergent cognitive patterns: VC cultures foster distinctive thinking styles that particularly benefit certain technology domains. As R. E. Nisbett et al. (2001) argued, cognitive traditions in these cultures prioritize fluency in convergent thinking, attention to structural relationships, and systematic categorization. Incidentally, these are all features applicable to technology design, systems architecture, and structured problem-solving approaches directly relevant to technology engagement, including Computational Thinking. Unlike more divergent thinking patterns that prioritize novel connections or contextual flexibility, these convergent cognitive approaches excel in domains requiring precise rule application, logical consistency, and systematic analysis, which are all skills highly valued in many traditional technology engagement pathways. It should be emphasized here that, while the framework discusses *tendencies* towards convergent and/or divergent cognitive patterns, successful technology engagement typically requires a dynamic interplay of *both* convergent (structured, synthesizing, and rule-based) and divergent (creative, oft-spontaneous, and contextual) thinking. Therefore, the current goal is to highlight cultural emphases in engagement modes, including the flexibility to navigate between convergent and divergent thinking patterns as appropriate, and not to imply fixed cognitive limitations or exclusive capacities across groups.

These priorities can serve to facilitate active technology engagement that aligns with success in both academic and professional settings in technology. VC cognition emphasizes the importance of foundational skills associated with orderly and convergent processes and structural understanding that directly support technology mastery, which can help explain why these cultural orientations often produce strong interest and achievement and representation across technology domains. These structured, expertise-aligned pathways often create high belongingness for VC learners, who experience technology mastery as a route to fulfilling culturally valued roles within hierarchical communities.

Horizontal Collectivist (HC) Technology Integration. In contrast to VC approaches, HC can foster different patterns in technology engagement that may not align as readily with traditional academic metrics but represent important cultural strengths:

1. Community-validated adoption: Technology adoption tends to spread through peer-and-community endorsement, rather than hierarchical channels such as formal education, in HC. This may create resistance to technologies perceived as imposed by external authorities without community validation (Cuban, 2003). Research by Moll et al. (2005) demonstrates how emerging technology adoption in HC communities often follows network-based diffusion patterns where trusted community leaders or members serve as innovation brokers and cultural translators. These adoption patterns create powerful authentic engagement when technologies resonate with community values but can create barriers when educational institutions fail to recognize or leverage these community-based validation processes. Unlike the hierarchical adoption characteristic of VC contexts described earlier, HC communities often develop sophisticated grassroots evaluation systems that prioritize community relevance over institutional endorsement, which is a process that educational systems frequently overlook or misinterpret as resistance rather than alternative validation.
2. Relationship-enhancing integration: Technologies tend to be valued in HC for their ability to strengthen community connections rather than establish specialized roles or tasks. Technology that ignores relational dimensions may therefore face resistance in HC (Collins, 2009). Research by Nasir and Hand (2008) illustrates how technology engagement in HC communities is frequently embedded within rich social contexts where the development of technological competence serves community relationships rather than existing as an independent pursuit. This orientation manifests in collaborative approaches to technology learning, where knowledge sharing becomes a form of social capital and technological expertise is valued for its contribution to collective endeavors. Unlike the role-specialization frameworks common in VC contexts, HC approaches often emphasize distributed expertise and reciprocal teaching relationships, where expertise is recognized but not hierarchically privileged in ways that might disrupt community equality.
3. Social equity emphasis: Research suggests that modern technologies are evaluated based on their contribution to advancing community welfare and addressing inequities in HC, as follows. Learners may disengage from technologies perceived as *perpetuating* rather than *rectifying* social inequalities (Scott et al., 2013). Further, Barton and Tan (2010) demonstrate how HC-oriented communities evaluate technological innovations through their potential to enhance community sovereignty and address historical inequities. This evaluative lens fundamentally shapes engagement with STEM fields, as learners assess whether technological pathways offer meaningful opportunities to address community challenges or merely reproduce existing power structures. Unlike the competence-focused evaluation criteria common in VC contexts, HC communities often apply what Eglash et al. (2013) point out as culturally relative criteria that emphasize how technologies might preserve cultural heritage while creating new opportunities for community advancement.

As seen, HC orientations do not resist technology itself but, rather, they may resist particular engagement modes, specifically surrounding technology education and training. Research by Watkins et al. (2018) found that Black and Latino U.S. youth demonstrate enthusiastic participation in technology *consumption*, from gaming, anime, to digital content creation, suggesting strong cultural resonance with technology as a medium for entertainment, interpersonal communication, and community building. This, incidentally, aligns with the first author’s own experiences in cultural spaces centered around technology consumption. From Little Tokyo in Los Angeles to the Akihabara district in Tokyo, he has consistently observed sizeable numbers of North American Black and Latino youth, alongside their Japanese and other Asian counterparts, enthusiastically engaging in vibrant participation as consumers of technology and digital culture, enjoying video games, anime merchandise, and digital collectibles, often in “cosplay.” These youth appear to demonstrate genuine engagement and cultural resonance with technology *products* as a means of sharing experiences. However, there is a paradox: although these community-based practices clearly demonstrate enthusiasm for technology-related products and activities, the forms of engagement in HC contexts appear to be situated primarily within consumption and communal participation rather than on technological production or innovation, as Watkins et al. have posited. In other words, this form of engagement, rooted in shared participation, does not necessarily seem to “translate” into robust pathways towards technological creation or leadership.

This, we contend, is not due to a lack of interest in technology as a field or even a sense of exclusion from the technology-based domains but, instead, perhaps reflects a deeper value alignment whereby HC cultures often prioritize shared experience, expressive participation, and community wellbeing over individual mastery or hierarchical advancement (Watkins et al., 2018), which may not align readily with the current methods of technology education and training. Specifically, mainstream STEM education and training pathways, and formal education in the U.S. in general, typically seem better aligned with the VC norms because they both emphasize expertise, structured assessment, achievement-based recognition systems, and navigation of knowledge without community contexts (Vélez-Ibáñez & Greenberg, 1992; Scott et al., 2013). This, unfortunately, may represent priorities that may feel culturally dissonant or even exclusionary for HC learners, according to the current framework. In sum, without *intentional* and *well-designed* bridges between relational, community-centered uses of modern technologies and the more formalized processes of technology engagement, we contend that many HC youth may remain engaged at the level of consumption but not production, despite their digital fluency. In sum, in HC communities, *belongingness* may best be nurtured when technologies support social connectivity and communal goals, making traditional individualistic pathways to technology engagement appear disconnected from culturally meaningful contributions.

Incidentally, the pattern of high engagement as consumers but underrepresentation as designers and producers also aligns with recent findings that Black students demonstrate strong interest in various modes technology but face substantial underrepresentation across technology fields which continue to grow (Hampton-Marcell et al., 2023). Similarly, despite Latino youth being early adopters of mobile technology and social media (Lopez et al., 2013), they remain significantly underrepresented in the technology workforce (Fry et al., 2021). The distinctive patterns of technology engagement across cultural value orientations can be systematically compared to highlight key differences in approaches, adoption processes, and evaluation criteria. Table 2 illustrates how belongingness may serve as a catalyst between cultural value orientations and corresponding technology engagement, showing how VC and HC frameworks may be associated with distinct belongingness experiences that match different patterns of technology participation and achievement.

The distinct patterns of “belongingness catalysts” shown in Table 2 highlight a critical opportunity: educational approaches that deliberately align with different cultural frameworks for experiencing belonging in technology spaces could potentially create more authentic and sustainable pathways to technology engagement across diverse communities of learners and professionals. Rather than suggesting that one set of groups (or cultural orientations) inherently values modern technologies more than another, these patterns indicate that belongingness in various “technology spaces” may be cultivated through fundamentally different pathways, some emphasizing hierarchy and structure, others, community integration. Notably, although HC individuals can experience belongingness as technology consumers, traditional pathways to technology production may not align with their cultural frameworks for achieving authentic belongingness, an opportunity explored in the following section.

**Figure 1:**
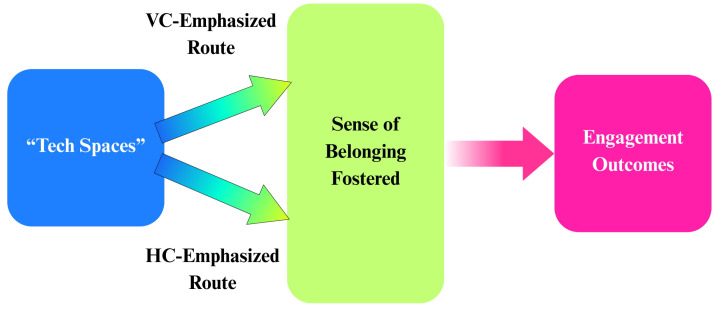
The BCVA Model. Technological spaces channel two culturally relative routes (vertical collectivism [VC], typically better aligned with technology education and training, and horizontal collectivism [HC], often less aligned with these structures) into belongingness, which in turn drives engagement outcomes (e.g., entry, persistence, advancement, and modes of participation such as consumption versus design).

These patterns suggest that the achievement gap reflects not disinterest in modern technologies but, rather, misalignment between culturally specific pathways to belongingness and traditional educational approaches that may inadvertently undermine authentic belonging for diverse learners. Educational interventions that foster belongingness through culturally aligned frameworks could leverage existing cultural strengths to create sustainable pathways to technology engagement. According to this proposed model, the Belongingness through Cultural Value Alignment (BCVA) model, developing effective educational approaches requires deliberately designing belongingness experiences that honor different cultural value orientations. Figure 1 illustrates the BCVA model, showing how technological engagement may be linked to cultural value priorities via belongingness.

To capture the detailed nuances of this model, Table 3 outlines the BCVA model’s belongingness-centered strategies that align with the distinct frameworks through which VC and HC communities experience authentic fit and acceptance, recognizing that cultural orientations represent assets for cultivating meaningful belonging rather than barriers to be overcome.

The BCVA-centered approaches in Table 3 are starting points for designing environments where diverse students can experience authentic belongingness aligned with their cultural frameworks. Many are readily actionable. For example, project-based learning, originating at McMaster University (Servant-Miklos, 2019), offers practical routes to HC-aligned belongingness, with age-appropriate adaptations. Likewise, COMPUGIRLS centers culturally responsive computing through community-rooted projects and peer-cohort validation; participants report feeling “seen” when work connects to collective narratives, illustrating the HC pathway in Table 3 (Scott et al., 2013). Building on this approach, Arizona State University’s CompuPower extends the model across high schools via a culturally responsive computing (CRC) course, mentor–teacher professional development, a multi-day residency, and parent workshops, documented by an independent evaluation (Clements et al., 2022). The design task is straightforward: embed both peer-cohort and expert-recognition cues so mixed cohorts can find authentic entry points to belongingness.

BCVA-informed approaches should be implemented alongside continued efforts to address structural barriers, including systemic bias and socioeconomic inequality. The most effective interventions integrate culturally grounded belongingness cultivation with ongoing structural reforms that expand access and opportunity. By attending to how cultural frameworks shape belongingness in technology spaces, this approach offers a pathway to psychologically grounded and culturally resonant strategies that address both structural barriers and belongingness needs.

## 3. Implications for Addressing Technology Achievement Gaps

Understanding how belongingness catalyzes technological engagement across cultural contexts provides new directions for addressing achievement gaps that move beyond *access-based* interventions to include *belongingness-centered* design. Formal U.S. educational approaches often welcome VC modes of technology engagement that align with the mainstream pedagogy and metrics while failing to recognize or build upon the strengths of HC orientations. Addressing technology achievement gaps requires recognizing both structural barriers and cultural assets concurrently, developing interventions that connect students’ cultural frameworks to educational pathways via fostering a sense of belonging, while addressing systemic inequities. Belongingness, therefore, should be seen as a core ingredient of culturally responsive education, not merely a precursor to engagement.

For HC contexts, effective approaches would unambiguously emphasize technology’s role in strengthening community connections and advancing social equity. Programs that embed technology education within community-centered projects addressing local issues have shown promise for engaging students (Watkins et al., 2018). Similarly, approaches that integrate family and community members into the learning process can leverage the cultural values priorities of the underrepresented groups to create more culturally resonant technology education (Lewis et al., 2019). This culturally attuned approach should be emphasized in designing technology teaching and learning, as it addresses the structural and cultural foundations of technology achievement gaps by developing interventions that work *with*, rather than *against*, authentic cultural frameworks that would promote a sense of belonging.

While Table 3 outlined BCVA-based belongingness-centered strategies for potential implementation, fostering authentic sense of belonging across cultural orientations may require coordinated intervention across the entire educational or professional ecosystem. Creating sustainable sense of belonging that serve as catalysts for technology engagement demands alignment, from policy level foci through educational and professional practice. Table 4 presents multi-level strategies for cultivating belongingness through the BCVA model, recognizing that authentic belonging cannot be achieved through isolated interventions but requires systemic commitment to honoring different pathways to experiencing fit and acceptance in technology spaces.

The multi-level strategies to optimize the sense of belonging, outlined in Table 4, demonstrate that creating authentic pathways to technology engagement requires coordinated efforts across educational and training systems, with each dimension reinforcing the belongingness experiences that ignite and sustain active participation as technology designers and producers rather than consumers. These strategies represent starting points for developing contextually appropriate approaches that cultivate belongingness through cultural alignment rather than imposing assimilation to dominant frameworks. Importantly, these belongingness-centered interventions must be developed collaboratively with the communities they aim to serve, ensuring that implementation reflects authentic cultural pathways to experiencing acceptance and fit rather than externally imposed assumptions about what belonging should look like. When educational systems commit to fostering belongingness as a catalyst for technological engagement, they create conditions where diverse students can experience the psychological foundation necessary for sustained participation and achievement.

## 4. Discussion and Limitations

This manuscript has introduced the BCVA model, a framework for understanding technology achievement gaps that positions belongingness as a critical catalyst between cultural value orientations and active engagement in technology research and development. By recognizing how VC and HC orientations create distinct belongingness experiences that drive technology engagement, educators, corporations, and policymakers can develop more effective interventions that foster authentic senses of belonging through culturally aligned pathways to belonging in the technology domains. Of course, as emphasized throughout this paper, this belongingness-centered lens should be applied alongside, not instead of, continued efforts to address systemic barriers such as discrimination, socioeconomic inequality, and inadequate resources. The most effective approaches will integrate belongingness fostering with ongoing structural reforms.

Because BCVA was developed in the United States, it should be treated as a heuristic lens, not a universal template. The BCVA model, organized around cultural value orientations, may nevertheless inform analyses of technology engagement and achievement gaps in other community, national, and cultural contexts. For example, persistent digital divides in parts of Europe and inequities in postcolonial settings could be examined through BCVA to ask how local orientations and institutional arrangements might cue belongingness. In the United Kingdom, scholars might study South Asian, Muslim, Caribbean-heritage, and other minoritized communities, while attending to local histories, definitions, and heterogeneity, to see whether particular validation cues foster or suppress a sense of belonging, especially in the technology spaces. Cross-national work indicates that cultural orientations and experiences of belongingness can take different forms across settings (Hornik & Tupchiy, 2006); therefore, international applications require local adaptation to historical and institutional contexts. In practice, this means co-designing with local stakeholders and validating constructs and measures in each setting before drawing inferences.

Similarly, researchers in East Asian educational systems might investigate how VC orientations create belongingness experiences differently across socioeconomic groups, genders, or various immigrant communities. We encourage international scholars to adapt, critique, and expand this BCVA framework through comparative studies that examine how belongingness, activated through cultural value orientations, might intersect with specific historical, economic, and social structures to lead to technology engagement patterns globally. Such dialogue would enrich our understanding of how belongingness experiences might explain technology engagement across populations and potentially lead to more contextually responsive interventions for a much wider range of groups.

To this end, future research should empirically examine the BCVA framework, investigating how cultural value orientations foster distinct belongingness experiences that catalyze technology engagement across populations and cultural contexts. Suitable study designs may include longitudinal surveys that trace how cultural value orientations predict changes in belongingness and, in turn, engagement; comparative ethnographies of classrooms that foreground different validation cues; and mixed-methods interventions that systematically vary peer-cohort validation and expert recognition. Belongingness should be modeled as a *mediating process* linking cultural orientations to engagement and measured with validated multi-item instruments adapted to computing contexts alongside behavioral indicators aligned with Table 3 (for example, peer affirmation and portfolio contributions in HC-emphasized designs, and milestone attainment and expert ratings in VC-emphasized designs). These studies should test whether interventions that foster culturally aligned belongingness produce stronger and more sustained engagement in technology spaces than those focused solely on access or accommodation. Policy implications include adopting belongingness-centered approaches to technology education and training that recognize and foster genuine belonging through distinct cultural pathways, rather than defaulting to standardized models that may suppress the sense of fit or acceptance needed for persistence. Adopting a BCVA-informed understanding of how cultural value orientations shape technology participation can support more effective strategies for addressing persistent achievement gaps across groups.

Finally, although belongingness ignited through alignment between cultural value orientations and technology education/training provides a promising lens for reimagining patterns of technology engagement, future research should examine how intersecting factors such as gender, socioeconomic status, regional variation, and evolving cultural practices interact with belongingness experiences. Such inquiries would help refine how different demographics and other characteristics may create unique belongingness conditions that shape technology engagement patterns in ways that merit dedicated research attention beyond cultural value orientations. For one, research indicates that participation patterns in technology fields vary significantly not only across cultural contexts but also across gender lines (Hayes, 2023), suggesting that belongingness experiences may be shaped by multiple intersecting identities. These considerations suggest that future studies should explore how cultural value orientations might create different belongingness pathways across intersecting identities, particularly examining how varying patterns of technology engagement is viewed, developed, and evaluated within different communities. Such research would complement the current BCVA framework, ultimately providing a more comprehensive understanding of how belongingness serves as a catalyst for technology engagement across diverse populations.

Along the same vein, it should be emphasized that, while cultural value orientations may provide insight into community-level belongingness patterns, this framework is not intended to deny the diversity of belongingness experiences, including individual differences, within any given group. Too often, discourses around minoritized populations emphasize only deficit-based perspectives, such as sociohistorical oppression, poverty, and linguistic mismatch, without recognizing how these communities may experience authentic belonging through their own cultural frameworks (Gouvea, 2021). This article aims to supplement such narratives by illuminating how cultural strengths create belongingness experiences that can serve as assets for fostering sustained technology engagement, rather than barriers to be overcome.

## 5. Conclusions

The Belongingness through Cultural Value Alignment (BCVA) model offers an outline for developing belongingness-centered approaches to technology education and training that recognize how distinct cultural orientations create distinct but equally valid pathways to experiencing fit and acceptance in technology spaces. Rather than attributing achievement gaps solely to structural barriers, educational approaches should leverage the belongingness experiences that may emerge from different cultural frameworks as catalysts for fostering active technological engagement, particularly in research and development, while simultaneously addressing the structural barriers that constrain opportunity. Educators and policymakers should support curriculum development that creates belongingness through various cultural pathways, professional development that helps educators recognize and cultivate students’ cultural assets as belongingness resources, and assessment approaches that validate diverse forms of technological competence as expressions of authentic belonging, while maintaining focus on dismantling the structural barriers that have historically limited opportunities for marginalized communities.

Most critically, the BCVA model contends that belongingness, when fostered through culturally aligned pathways that may resonate with learners from a wider range of backgrounds, may serve as the key psychological mechanism that could transform cultural orientations into sustained and effective engagement with technology domains among them. We propose that achievement gaps may persist, not exclusively because of limited access or exclusion but also because educational environments often fail to cultivate the belongingness conditions needed to spark engagement across varying cultural frameworks. In this sense, the BCVA framework offers design-relevant guidance for fostering active engagement in education and training in such contexts as emerging technologies and computational thinking—in ways that resonate with learners from a variety of cultural orientations. Such approaches can better prepare currently underrepresented groups for future technology careers. By designing technology spaces to cultivate belongingness among all learners, we may open effective and equitable pathways across the ecosystem of modern technologies, from computational thinking, coding and design, data and AI practice, security and governance to platform co-creation and governance.

## Figures and Tables

**Table 1:** Proposed Cultural Frameworks for Experiencing Belongingness in Technology Spaces.

Dimensions	Vertical Collectivism (VC)	Horizontal Collectivism (HC)
Key Belongingness Pathway	Recognition vis-a-vis hierarchical structures through demonstrated expertise and structured achievements	Community integration through peer support, collective contribution, and mutual validation
Group Structure	Structured hierarchies with established roles that provide clear pathways to recognition	Flattened authority structures with distributed responsibilities that emphasize equality
Decision-making Process	Deference to established authority and expert figures validates belonging through expert acknowledgment	Consensus-building through community dialogue creates belonging through inclusive participation
Knowledge Transmission	Structured pathways following hierarchy of expertise create belonging through progressive mastery	Informal and lateral networks create belonging through reciprocal learning relationships
Individual–Group Relationship	Personal goals align with group goals within hierarchical framework, with belonging earned through role fulfilment	Personal goals align with group welfare, with belonging achieved through community contribution
Achievement Recognition	Formal recognition within established hierarchies validates technological competence and belonging	Community acknowledgment of contributions to collective welfare affirms belonging and value
Experiences That Promote Belonging	Expert validation, skill mastery acknowledgment, hierarchical advancement, structured milestone recognition	Community acceptance, peer affirmation, collective impact recognition, mutual support validation

**Table 2:** Belongingness-Driven Technology Engagement Patterns.

Dimensions	Vertical Collectivism (VC)-Emphasized	Horizontal Collectivism (HC)-Emphasized
Primary Belongingness Strategy	Achieve belonging through expert validation of technological competence within established hierarchies	Achieve belonging through technology’s ability to foster community connections and advance collective welfare
Technology Positioning for Belongingness	Role- or task-based integration within existing hierarchical structures that include both humans and technology	Relationship-enhancing tools valued for their ability to build community bonds and solidarity
Learning Pathway to Belongingness	Expert-sanctioned, structured mastery progression that leads to expert recognition and hierarchical advancement	Community-validated adoption through peer networks that emphasizes shared experience and mutual learning
Cognitive Approach Supporting Belongingness	Convergent thinking emphasizing structural relationships and systematic categorization that aligns with the demands of the task at hand	Contextual thinking emphasizing social applications and community benefits that resonates with peer values
Evaluation Criteria for Belongingness	Technological proficiency and competence development measured against established standards	Social equity impact and community contribution measured through collective benefit and peer validation
Belongingness Validation Process	Expert-sanctioned learning through established hierarchies leading to formal recognition	Community-validated adoption through peer networks leading to collective endorsement
Observable Belongingness Outcomes	Systematic skill development, hierarchical role achievement, and expert acknowledgment	Enhanced community connections, collaborative innovations, collective problem-solving contributions

**Table 3:** The BCVA Model-Informed Design Principles for Technology Education and Training.

Design Element	To Foster Primarily VC-Emphasized Belongingness	To Foster Primarily HC-Emphasized Belongingness
Target Belongingness Experience	Experience authentic belonging through expert recognition of their technological competence and structured achievement within clear hierarchies	Experience authentic belonging through peer validation of their contributions to community-centered technological solutions
Instructional Structure for Belongingness	Progression with achievement milestones that provide clear pathways to expert recognition	Community-embedded projects addressing local issues that create opportunities for peer validation and collective impact
Expert Relations Supporting Belongingness	Clear expert–learner roles that validate learning through expert leadership and validation	Collaborative learning communities with distributed expertise that affirm belonging through peer recognition
Assessment Approaches for Belongingness	Competency-based evaluations aligned with industry standards that provide expert validation of technological mastery	Portfolio assessments showcasing community contributions that enable peer evaluation and collective validation
Motivational Framework for Belongingness	Recognition within established hierarchies through knowledge and skill achievement that fulfills their roles	Contribution to community wellbeing and social equity that demonstrates value through collective impact
Family/Community Integration for Belongingness	Parent education about educational pathways that validates family or community investment in achievement	Active family and community participation as co-designers that affirms cultural values in learning process
Technology Selection for Belongingness	Tools emphasizing systematic skill development that align with expert expectations and structured competence building	Technologies supporting communication, collaboration, and community problem- solving that enhance relational connections
Belongingness Success Indicators	Expert validation received, skill milestones achieved, hierarchical progression demonstrated, structured recognition obtained, etc.	Community acceptance gained, peer affirmation received, community impact achieved, mutual support relationships established, etc.

**Table 4:** Multi-level Strategies for Fostering Belongingness in Technology Education a la BCVA.

Level of Intervention	To Promote Vertical Collectivist (VC)- Aligned Belongingness	To Promote Horizontal Collectivist (HC)- Aligned Belongingness
Educational Policy	Hierarchical pathways with clear skill mastery milestones and recognition that enable expert validation and structured belonging	Community-based technology initiatives and flexible credentialing that affirm collective ownership and peer validation
Curriculum Design	Sequential skill development with explicit connections to established knowledge and skill hierarchies that foster belongingness through expert recognition and incremental mastery	Project-based learning addressing community challenges with direct community impact that foster belongingness through collective contributions
Teaching Methods	Expert-guided instruction that validates belong through incremental knowledge/skill mastery and authority acknowledgment	Collaborative learning communities with distributed expertise and peer mentoring that affirm belonging through mutual recognition
Assessment Approaches	Competency-based evaluations aligned with standards that provide expert validation and belonging confirmation	Portfolio assessments showcasing community contributions and collaborative achievements that enable peer validation and collective belong
Family Engagement	Family education about educational pathways and career trajectories that validates family investment in hierarchical achievement and belonging in technology	Active family participation as co-designers and contributors to learning experiences that affirms cultural values and community belonging
Technology Selection	Tools emphasizing systematic skill development and technological proficiency that align with expert expectations and structured belonging pathways	Technologies supporting communication, collaboration, and community problem-solving that enhance relational connections and collective belonging
Structural Support	Mentoring from experts that provide pathways to validation and hierarchical belonging	Community technology centers with local or institutional leadership and intergenerational programming that foster peer validation and collective belonging

## Data Availability

No new data were created or analyzed in this study. Data sharing is not applicable to this article.
